# Metabolic adaptation following gastric bypass surgery: results from a 2-year observational study

**DOI:** 10.1038/s41366-024-01585-5

**Published:** 2024-09-03

**Authors:** Fathimath Naseer, Shu-Dong Zhang, Alexander D. Miras, Tamsyn Redpath, Melanie Martin, Adele Boyd, Heather Spence, Dimitri J. Pournaras, Zsolt Bodnar, David Kerrigan, Carel W. le Roux, M. Barbara E. Livingstone, Ruth K. Price

**Affiliations:** 1 Nutrition Innovation Centre for Food and Health (NICHE), Ulster University, Coleraine, BT52 1SA United Kingdom; 2 School of Medicine, Ulster University, Londonderry, BT48 7JL United Kingdom; 3 Department of Bariatric and Metabolic Surgery, North Bristol NHS Trust, Southmead Hospital, Bristol, BS10 5NB England; 4 Department of Surgery, Letterkenny University Hospital, Donegal, Ireland; 5 Phoenix Health, 17E-F Telford Court, Chester, CH1 6LT England; 6 Diabetes Complications Research Centre, Conway Institute, University College Dublin, Dublin, Ireland

**Keywords:** Obesity, Weight management

## Abstract

**Background/Objectives:**

Metabolic adaptation is the lowering of basal metabolic rate (BMR) beyond what is predicted from changes in fat mass (FM) and fat-free mass (FFM) and may hamper weight-loss progression. It is unclear whether metabolic adaptation occurs following gastric bypass surgery (GBP) and if it persists. The aim of this study was to evaluate the reduction in BMR that is not explained by changes in body composition in patients following GBP compared to a weight-stable comparator group.

**Subjects:**

Thirty-one patients [77.4% female; mean BMI 45.5(SD 7.0) kg/m^2^; age 47.4(11.6)y] who underwent GBP, and 32 time-matched comparators [50% female; BMI 27.2(4.6) kg/m^2^; age 41.8(13.6)y) were evaluated at 1-month pre-surgery, 3-, 12- and 24-months post-surgery.

**Methods:**

BMR was measured under standardised residential conditions using indirect calorimetry and body composition using DXA. Linear regression analyses assessed metabolic adaptation post-surgery.

**Results:**

After surgery, patients lost a quarter of their body weight [−25.6%(1.8%); *p* < 0.0001] consisting mainly of FM (4:1 FM to FFM loss ratio) at 24-months post-surgery. Absolute BMR (MJ/d) reduced by 25.7% at 24-months post-surgery with values becoming similar to the comparator group from 3-months post-surgery. Positive associations were observed between changes in BMR and changes in FFM and FM (*P* < 0.03). Metabolic adaptation was present in patients during the 1) rapid weight loss phase (6.9 kg/month at 3-months post-surgery) (*p* = 0.011), 2) slower weight loss phase (1.6 kg/month from 3 to 12-months post-surgery) (*p* < 0.0001), and, 3) weight maintenance phase (24-months post-surgery) (*p* = 0.00073). However, the degree of metabolic adaptation observed in GBP patients was similar to the weight-stable comparator group (no metabolic adaptation) from 12-months post-surgery onwards (3-months; *p* = 0.01, 12-months; *p* = 0.26, 24-months post-surgery; *p* = 0.70).

**Conclusion:**

These results suggest that there is a potential biological mechanism of surgery that attenuates the expected postoperative downregulation in BMR thus helping GBP patients maintain weight loss.

## Introduction

Gastric Bypass Surgery (GBP) is one of the most frequently performed bariatric surgeries [1]. It yields significant weight loss of approximately 60–70% of excess body weight at 1-year post-surgery with improvements in associated health complications and quality of life outcomes [2]. There are multiple mediators involved in weight loss following GBP that cannot be explained by restrictive and malabsorptive mechanisms [3, 4]. Identifying such mediators is essential for both advancing current understanding of the biology of obesity and providing evidence-based clinical advice for managing weight loss and related clinical outcomes [5].

During negative energy balance and weight loss, metabolic adaptation leads to downregulation in measured BMR that cannot be explained exclusively by a reduction in fat-mass (FM) and fat-free mass (FFM) [6–8]. It is thought to favour resistance to weight loss and contribute to weight recividism [9]. In a retrospective study of metabolic adaptation, individuals who lost weight through non-surgical interventions exhibited a higher degree of metabolic adaptation (at 7-months post-intervention) compared to individuals after GBP (at 6-months post-surgery) [10]. Another observational study noted that despite GBP patients losing significantly more weight and lean body mass than gastric banding patients, the degree of metabolic adaptation between groups was similar at 6-months post-surgery [11]. Taken together, these data suggest a potential biological mechanism that may help explain, at least in part, the substantial and sustained weight loss after GBP. Additionally, it has been demonstrated that the observed metabolic adaptation dissipated at follow-ups of >6 months post-surgery [10, 12, 13]. However, no study to date has evaluated longer term (≥2 years) changes in metabolic adaptation following GBP.

Therefore, the aim of this study was to evaluate the prospectively measured reduction in BMR that is not explained by changes in FM and FFM in patients undergoing GBP and compare with a non-surgical weight stable comparator group at 3-, 12- and 24-months post-surgery. It was hypothesised that the expected down regulation of BMR observed after weight-loss, over and above that explained by changes in FM and FFM, would be attenuated in patients by 3-month post -surgery.

## Materials/subjects and methods

### Study design

The hypothesis for this paper was tested as a secondary hypothesis within a wider study investigating changes in energy intake following GBP, which is described in detail elsewhere [14, 15]. Owing to the novel study protocol, the sample size was estimated from the patient population recruited for a randomised controlled trial [16] that detected significant differences in self-reported energy intake between Vertical Banded Gastrectomy (VBG (*n* = 7) and GBP (*n* = 9) participants at 6 years post-surgery. The SD associated with the change in dietary fat intake (% energy) from pre- to post-surgery and a 95% confidence interval was applied as follows:

$$n={\left(\frac{{confidence\; level}\times{SD}}{{margin\; of\; error}}\right)}^{2}$$

$$n={\left(\frac{{1.96}\,\times\,{1.9}}{1}\right)}^{2}$$

$$n=14$$

It was estimated that at least 16 participants are required for the present study based on a 14% attrition rate reported by another similar intake study [17]. However, as the proposed study protocol was intensive for study participants, 32 GBP patients and a similar number of weight-stable comparators were recruited to account for a potentially higher attrition rate.

In brief, in this study, all participants were required to complete four fully residential study assessments at 1-month pre-surgery, 3, 12- and 24-months post-surgery at the Human Intervention Studies Unit (HISU) at Ulster University. HISU consists of en-suite bedrooms, a communal sitting room, a metabolic kitchen (closed access to participants) and communal dining room. Participants arrived at HISU at approximately 6 pm on day 1 for an initial acclimation period where no measurements were performed. Following a standard meal of Spaghetti Bolognese for dinner (if requested), participants fasted from 10 pm. Measurements started early on day 2 (approx. 7am) and lasted until bedtime (approx. 11 pm) on day 2. Participants remained sedentary throughout but were free to engage in light activities such as reading, crafts and watching television.

Patients were recruited from four sites in the United Kingdom (Phoenix Health NHS, Phoenix Health Private, London Imperial Weight Centre and North Bristol NHS Trust) and one site in the Republic of Ireland (Letterkenny University Hospital). Inclusion criterion were ≥18 years old with a scheduled GBP. Weight-stable (>6 months) comparators were time-matched and recruited by posters, email circulations, radio, and social media platforms. The purpose of the comparator group was to account for external factors which could potentially impact GBP patients over the study period, as well as any change in behaviour in the residential unit over the four time points. Inclusion criterion were ≥18 years old with no plans to alter current body weight. Exclusion criteria for all participants were: presence of physical or psychological conditions affecting food intake; strict dietary restrictions, food allergies and pregnant or lactating women.

Total body weight, body mass index (BMI), FM, FFM, visceral adipose tissue (VAT) and subcutaneous adipose tissue (SAT) were assessed under standardised conditions using the total body GE Lunar iDXA scan (GE Healthcare, USA). Height was measured during the initial visit to the nearest 0.1 cm using a wall-mounted stadiometer (Seca Ltd, Hamburg, Germany (%CV = 0.23%). If the participant’s body width exceeded the standard dimension of the DXA’s scanning area, they were positioned such that the right half of the body was fully within the scan field. Half scans have shown satisfactory validity [18]. DXA measurements were conducted by trained researchers and verified by a qualified health care professional.

Percentage total weight loss (%TWL) was calculated as$\% {TWL}=\frac{\left({weight\; prior\; to\; surgery}-{follow}-{up\; weight}\right)}{{weight\; prior\; to\; surgery}}\,x\,100$. Postoperative weight loss was also expressed as a percentage excess of weight loss (%EWL) following the formula:${EWL}=\frac{({\rm{weight\; prior\; to\; surgery}}-{\rm{follow}}-{\rm{up\; weight}})}{({\rm{weight\; prior\; to\; surgery}}-{\rm{weight\; corresponding\; to\; BMI}}=25{\rm{kg}}/{{\rm{m}}}^{2})}x100$.

BMR was measured under standardised conditions following an overnight fast (from 10 pm) using open-circuit portable indirect calorimetry (ECAL, Metabolic Health Solutions) by a trained researcher. Each participant was awakened at approximately 7am in the morning to empty their bladder and return to rest for at least 30 min in a quiet, darkened and thermoneutral room before the measurement was made. Distractions such as use of mobile phones were not permitted. Data were recorded for a minimum of 8-minutes and was terminated after readings had been stable for 45 s. The first 2-minutes of the measurement period were automatically discarded by the ECAL software, with any other anomalous recordings (e.g., coughing, removal of mouthpiece) also discarded as ‘false’ readings. BMR values were calculated using the Weir formula [19].

To determine the magnitude of metabolic adaptation following GBP, this study used the gold standard methodology [6, 7, 10, 11, 20, 21]. The baseline BMR (dependent variable) for both patient and comparator groups was used to generate a linear regression model with multiple predictor variables (independent variables) that may affect BMR values - baseline FM, FFM, age, gender, medications, group (participants) and medical conditions. This model was used to predict the BMR (pBMR) at 3-,12- and 24-months post-surgery.

$$\begin{array}{lll} &&{\rm{pBMR}}\left({\rm{MJ}}/{\rm{day}}\right)\\ &&\quad =3.529-(1.509\,\times\,{\rm{Participants}})+(0.511\,\times{\rm{Gender}})\\ &&\qquad -\,(0.001\,\times\,{\rm{Age}}\; {\rm{in}}\; {\rm{years}})+(0.022\,\times\,{\rm{FM}}\; {\rm{in}}\; {\rm{kg}})+(0.088\,\times\,{\rm{FFM}}\; {\rm{in}}\; {\rm{kg}})\\ &&\qquad +\,(0.936\,\times\,{\rm{Medication}}\; {\rm{that}}\; {\rm{affect}}\; {\rm{BMR}})-(0.513\,\times\,{\rm{Disease}}\; {\rm{that}}\; {\rm{affect}}\; {\rm{BMR}})\end{array}$$

*Participants (1 for Patient, 2 for Comparator), Gender (1 Female, 2 Male), Medications that affect BMR (1 Prescribed, 2 Not prescribed), Diseases that affect BMR (1 Present, 2 Absent)*.

Finally, the residual BMR (resBMR) is defined as the difference between the observed BMR (as measured by indirect calorimetry) from the predicted BMR based on the above linear regression equation.

$${BMR\; residual}=({\rm{measured\; BMR}}-{\rm{predicted\; BMR}})$$

And the presence of metabolic adaptation is defined as resBMR being significantly different from zero.

### Statistical analysis

Statistical analyses were performed using IBM SPSS for windows (UK, version 26.0) and R (version 4.2). Baseline summary statistics are expressed as mean (SD) for continuous variables, or as numbers (percentage) for categorical variables. Results from linear mixed models were presented as least squares mean (SEM).

**Fig. 1:**
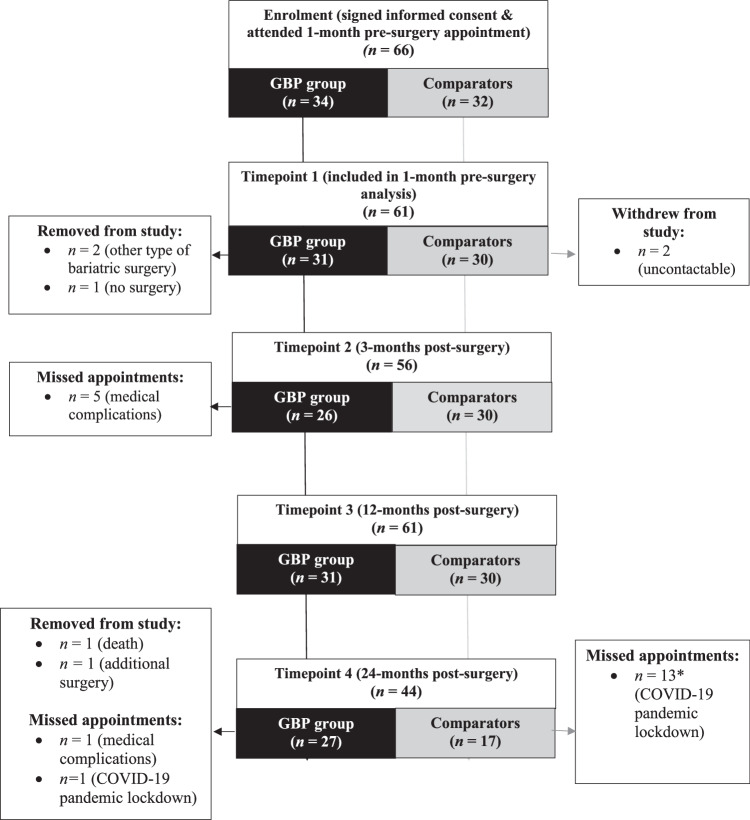
Participant attendance through the four time points of the Gastric Bypass Study. *Comparators not follow-up during pandemic lockdown. GBP Gastric Bypass Surgery.

At each time-point, there were some random missing values due to missed appointments (Fig. 1) and, in a few cases, technical issues with measuring equipment. Given that it is reasonable to assume that such values were missing purely at random, mixed effects linear models were fitted for the main outcome measures of interests (Weight, BMI, FM, FFM, LBM, VAT, SAT, and BMR). In each of these linear mixed models, participant IDs were fitted as random effects, with participant group (patient or comparator), time and the interaction between group and time as fixed effects. For such linear mixed modelling, no imputation of missing values was conducted as this was unnecessary. The same linear mixed modelling approach was applied to residual BMR (resBMR), where baseline weight was included as a covariate to account for its potential effects on metabolic adaptation, From the fitted linear mixed models, the estimated means and standard errors of the outcome measure were then obtained for all group and time point combinations. Where applicable and deemed interesting, comparative analysis between different time points per group, or between the two groups per time point were conducted by testing the corresponding general linear hypothesis using Z-test and the single-step method for multiple testing adjustment.

Mixed model analysis was applied to the residual BMR using the single-step method for multiple testing adjustments to determine the presence or absence of metabolic adaptation. Based on the parameters of the fitted linear mixed model for residual BMR, the presence or absence of metabolic adaptation was determined by testing the corresponding general linear hypothesis. Metabolic adaptation was considered to have occurred if BMR residual (magnitude of metabolic adaptation) was significantly different from zero (*p* ≤ 0.05). Pearson correlation coefficients were used to study associations between changes in FM, FFM, %FFM/ weight and BMR in patients, and extent of metabolic adaption and weight loss. *P*-values of ≤0.05 were considered as statistically significant.

### Ethics approval and consent to participate

Ethical approval was granted by the West of Scotland Research Ethics Service (WoSRES) (REC 16/WS/0056, IRAS 200567). The study was registered as a clinical trial (NCT03113305) (clinicaltrials.gov) and was conducted according to the principles of the Helsinki declaration. The primary outcome of the work was changes in dietary energy intake, with the work presented in this study included as secondary outcomes. Prior to the start of the study written informed consent was obtained from all participants.

## Results

Sixty-six participants attended the baseline study appointment (Fig. 1). Three of the patients were subsequently excluded as they did not receive GBS (Sleeve Gastrectomy surgery, *n* 2; medical issues, *n* 1). Of the remaining 63 participants, two individuals from the comparator group were uncontactable after the first appointment, leaving 31 patients and 30 comparators (Fig. 1). Following the COVID-19 pandemic lock down period only patients were followed up for their final 24-month appointment.

The patient group had a higher proportion of females and were more likely to present with diabetes mellitus pre-surgery.

### Body composition

A reduction in all anthropometric variables was observed in patients by 3-months post-surgery, with stability in changes from pre-surgery achieved at 12- and 24-months (Table 1). Over the 24-month study period patients had lost over a quarter of their total mean weight −25.6% (SD 1.8)% from pre-surgery (*P* < 0.001), while the comparator group remained weight-stable throughout the study period (*p* = 0.96).

**Table 1:** Mean anthropometric measures and basal metabolic rate at baseline (1-month pre-surgery) and at 3-, 12- and 24-months post-surgery.

	1-month pre-surgery	3-months post-surgery	12-months post-surgery	24-months post-surgery	ANOVA P values for
Weight (kg)					Group <0.0001
Patients	122.90(3.08)	102.31 (3.13)	87.92 (3.08)	89.86 (3.13)	Time <0.0001
Comparator group	78.01 (3.13)	78.25 (3.14)	78.63(3.13)	79.78 (3.34)	Group:Time <0.0001
BMI (kg/m^2^)					Group <0.0001
Patients	45.47 (0.92)	37.81 (0.94)	32.38 (0.92)	33.06 (0.94)	Time <0.0001
Comparator group	27.22 (0.94)	27.25 (0.95)	27.39 (0.94)	27.64 (1.03)	Group:Time <0.0001
Fat mass (kg)					Group <0.0001
Patients	62.07 (2.00)	46.78 (2.06)	33.73 (2.00)	35.98 (2.06)	Time <0.0001
Comparator group	26.17 (2.04)	26.55 (2.05)	26.62 (2.04)	27.00 (2.26)	Group:Time <0.0001
Fat-free mass (kg)					Group 0.14
Patients	60.83 (1.90)	55.54 (1.91)	54.19 (1.90)	53.84 (1.91)	Time <0.0001
Comparator group	51.84 (1.94)	51.70 (1.94)	52.01 (1.94)	52.80 (1.96)	Group:Time <0.0001
LBM (kg)					Group 0.13
Patients	58.04 (1.83)	52.79 (1.83)	51.52 (1.83)	51.09 (1.83)	Time <0.0001
Comparator group	49.13 (1.86)	48.99 (1.86)	49.30 (1.86)	50.10 (1.89)	Group:Time <0.0001
VAT (kg)					Group 0.018
Patients	3.10 (0.19)	1.93 (0.20)	1.29 (0.19)	1.20 (0.20)	Time <0.0001
Comparator group	1.00 (0.20)	1.02 (0.20)	1.06 (0.20)	1.05 (0.21)	Group:Time <0.0001
SAT (kg)					Group <0.0001
Patients	58.96 (1.91)	44.85 (1.97)	32.44 (1.91)	34.78 (1.97)	Time <0.0001
Comparator group	25.17 (1.94)	25.53 (1.95)	25.55 (1.94)	25.95 (2.15)	Group:Time <0.0001
BMR (MJ/day)					Group 0.0077
Patients	9.93 (0.38)	7.72 (0.41)	7.18 (0.37)	7.38 (0.39)	Time <0.0001
Comparator group	7.03 (0.40)	7.52 (0.38)	6.52 (0.39)	6.57 (0.46)	Group:Time <0.0001

Data presented as mean (SE) based on the linear mixed model estimate. Patients *n* 31, *n* 26, *n* 31, *n* 26; Comparators *n* 30, *n* 29, *n* 30, *n* 17 for 1-month pre-surgery, 3-, 12 and 24-months post-surgery respectively. In each of these linear mixed models, participants were fitted as random effects, while group, time and the interaction between group and time as fixed effects. For all the variables listed in this table, from Weight to BMR, ANOVA tests suggest that the interaction between Group and Time is highly significant, meaning that the difference between the two Groups depends on Time. (The time trajectory curves for the two groups are far from parallel based on the linear mixed models, see Supplementary figures).

*BMI* Body Mass Index, *BMR* Basal Metabolic Rate, *LBM* Lean Body Mass, *SAT* Subcutaneous Adipose Tissue, *VAT* Visceral Adipose Tissue.

At 24-months post-surgery 71% (*n* = 22) of patients had achieved successful weight loss (>50%EWL), with three patients regaining weight (<50%EWL) and two patients continuing to lose weight (achieving >50%EWL) from their 12-month measurement.

**Fig. 2:**
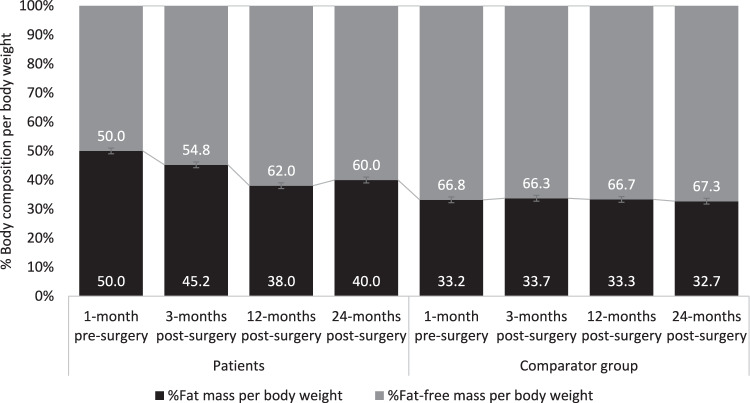
Mean %proportion of fat mass and fat-free mass per bodyweight for participants at 1-month pre-surgery and at 3-, 12- and 24-months post-surgery. Data presented as arithmetic mean (SEM) of data available per group at each time point. n: (-1 month pre-surgery: patients 31, comparators 30, 3-months post-surgery: patients 26, comparator 29, 12-months post-surgery: patients 31, comparators 30, 12-months post-surgery: patients 26, comparator 17).

The majority of weight loss following GBP was accounted for by a decrease in FM. On average, patients lost 40% of pre-surgery FM and 11% of pre-surgery FFM at 24-months post-surgery, a ratio of roughly 4:1 FM to FFM in terms of percentage loss. By 3 months post-surgery FFM was similar in both the patient and comparator groups, with *p*-values being 0.68, 0.97, and 1.0 respectively for Month 3, 12 and 24 post surgery. On the other hand, comparing patient versus comparator groups in terms of FM, the *p*-values were <0.0001 for 3-months, 0.10 for 12-months, and 0.028 for 24-months post-surgery. Therefore, patients’ FM largely remained higher than comparators and as a result, the mean total body %FM decreased at each timepoint from 50.0% pre-surgery to 40.0% 24-months after surgery (Fig. 2).

### Basal metabolic rate

In the linear mixed modelling for all outcome variables from weight to BMR, ANOVA tests suggest that the interaction between group and time is highly significant – meaning that the difference between groups depend on time. The time course curves of the two groups are therefore not parallel (See Supplementary Figures). Based on the estimates from the linear mixed models, absolute BMR (MJ/d) was 29% higher in patients than the comparator group pre-surgery [9.93(0.38) *vs* 7.03(0.40) MJ/d for patients and comparator group respectively, *p* < 0.0001] but was similar to the comparator group at all post-surgery time-points despite a significant reduction in BMR post-surgery (<22%). Absolute BMR values remained stable in the comparator group at all three post-surgery assessments in comparison to pre-surgery (<+/−7%) (*p* = 0.86, 0.86, and 0.94 respectively).

**Fig. 3:**
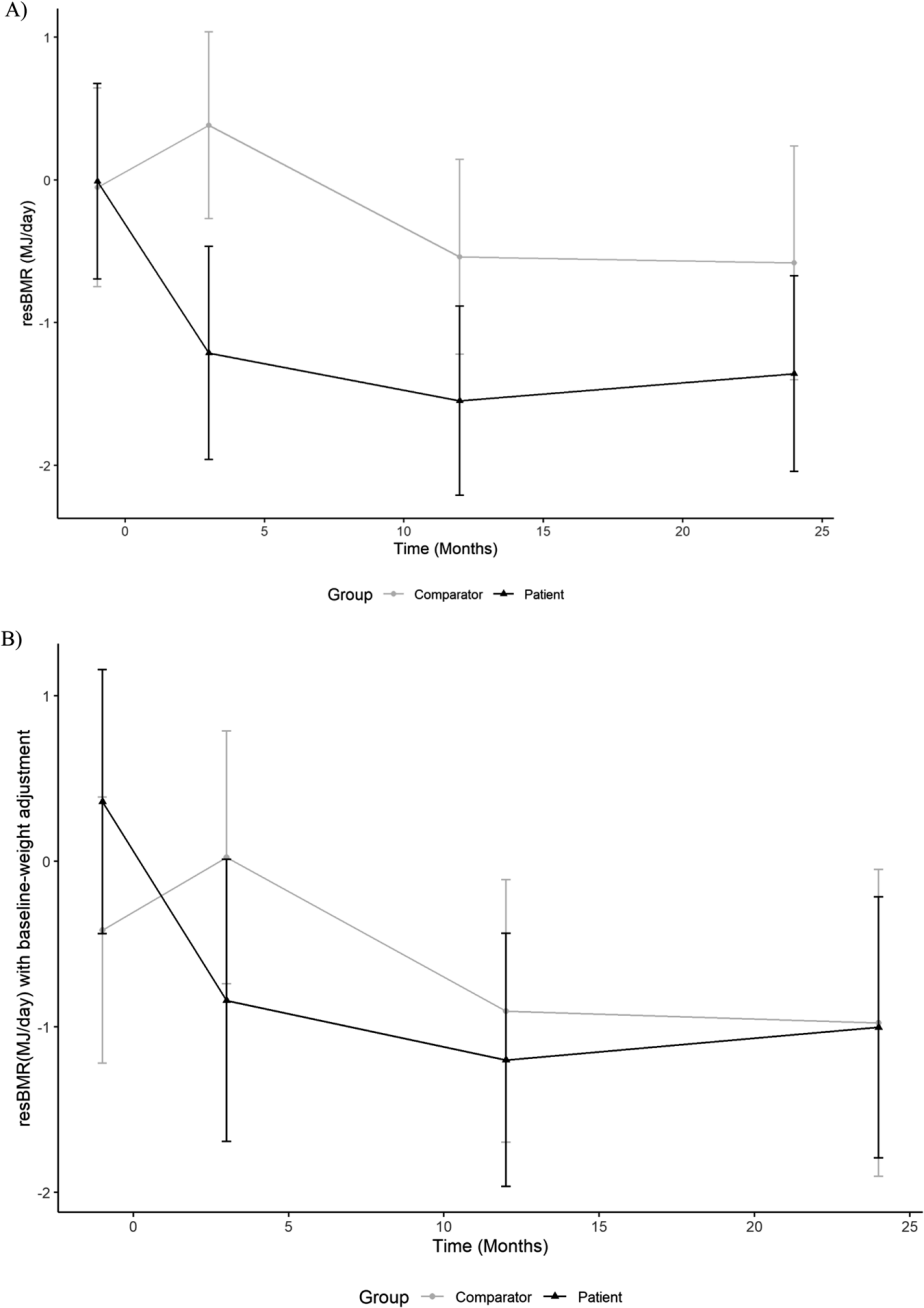
Time trajectory plots of residual BMR (resBMR) or magnitude of metabolic adaptation for patient and comparator groups. Time trajectory plots of resBMR (**A**) and baseline-weight adjusted resBMR (**B**) for patient and comparator groups before and 3-, 12- and 24-months after Gastric Bypass Surgery. Errors represents the 95% confidence intervals of the estimated means based on the fitted linear mixed model. resBMR is defined as the difference between the observed BMR (as measured by indirect calorimetry) from the predicted BMR based on the linear regression equations. **A** Difference between groups: 1-month pre-surgery (*p* = 1.00), 3-months post-surgery (*p* = 0.014), 12-months post-surgery (*p* = 0.26) and 24-months post-surgery (*p* = 0.70). **B** Difference between groups:1-month pre-surgery (*p* = 0.84), 3-months post-surgery (*p* = 0.76), 12-months post-surgery (*p* = 1.0) and 24-months post-surgery (*p* = 1.0).

In the linear mixed modelling analysis for resBMR (the metabolic adaptation measure), ANOVA test indicated that the group time interaction is at the margin of statistical significance (*p* = 0.052) - suggesting that a simpler model without the group time interaction could be used. However, a log likelihood ratio test comparing the models with and without the interaction, indicated that the full model (with interaction) is still better than that model without the interaction (*P* = 0.045). Therefore, for the measure of metabolic adaptation, the full model was retained to obtain the least square means and standard errors for all the group time point combinations. Based on the obtained linear mixed model, the hypotheses regarding whether resBMR estimate at each time point per group is different from 0 (*p* ≤ 0.05) were tested as shown in Fig. 3. Metabolic adaptation was present post-surgery for patients only (*p* = 0.011 at 3 months post -GBP; *p* < 0.0001 at 12-months and *p* = 0.00073 at 24-months post-surgery.

Figure 3 Time trajectory plot showing the degree of metabolic adaptation for patients compared to comparators at 3-months, 12-months and 24-months following GBP. A significant difference between groups was noted at 3-months post-surgery (*P* = 0.014) and the degree of metabolic adaptation was similar between groups pre-surgery (*p* = 1.00), 12-months post-surgery (*p* = 0.26) and 24-months post-surgery (*p* = 0.70).

In patients, a positive correlation was observed between changes in FFM (kg) and changes in absolute BMR at 12-months (r = 0.45, *p* = 0.025) and 24 months post-surgery (r = 0.*50*, *p* = 0.012) (Table 2). Similarly, a positive correlation was observed between changes in %FFM/weight and changes in absolute BMR at 12-months (r = 0.50, *p* = 0.010) and 24-months post-surgery (r = 0.50, *p* = 0.011). Changes in FM (kg) and changes in absolute BMR were positively correlated at 12-months post-surgery (r = 0.65, *p* = 0.00041) and 24-months post-surgery (r = 0.64, *p* = 0.00053). No other associations were observed.

**Table 2:** Associations between changes in body composition and basal metabolic rate for patients and comparator groups at 3-months, 12-months and 24-months post gastric bypass-surgery.

	3-months post-surgery		12-months post-surgery		24-months post-surgery	
	Patients (*n* 20)	Comparator group (*n* 24)	Patients (*n* 25)	Comparator group (*n* 21)	Patients (*n* 25)	Comparator group (*n* 17)
	ΔBMR (MJ/day)		ΔBMR (MJ/day)		ΔBMR (MJ/day)	
ΔFM (kg)	0.20 (95%CI: −0.27 to 0.59; *p* = 0.40)	0.23 (95% CI: −0.19 to 0.58; *p* = 0.28)	0.65^a^ (95% CI: 0.35 to 0.83; *p* = 0.00041)	0.25 (95% CI: −0.21 to 0.61; *p* = 0.28)	0.64 ^a^ (95% CI: 0.33 to 0.83; *p* = 0.00053)	0.04 (95% CI: −0.45 to 0.51; *p* = 0.89)
ΔFFM (kg)	0.33 (95% CI: −0.14 to 0.67; *p* = 0.16)	−0.20 (95% CI: −0.56 to 0.23; *p* = 0.36)	0.45 ^a^ (95% CI: 0.06 to 0.72; *p* = 0.025)	−0.13 (95% CI: −0.53 to 0.32; *p* = 0.60)	0.50^a^ (95% CI: 0.13 to 0.74; *p* = 0.012)	−0.02 (95% CI: −0.49 to 0.47; *p* = 0.94)
Δ%FFM/weight kg	−0.11 (95% CI: −0.53 to 0.35; *p* = 0.64)	0.24 (95% CI: −0.18 to 0.58; *p* = 0.27)	0.50 ^a^ (95% CI: 0.13 to 0.75; *p* = 0.010)	0.26 (95% CI: −0.20 to 0.62; *p* = 0.26)	0.50 ^a^ (95% CI: 0.13 to 0.75; *p* = 0.011)	0.09 (95% CI: −0.41 to 0.55; *p* = 0.72)

Associations analysed using Pearson’s correlation. Data presented as r (correlation coefficient) with 95% confidence interval (CI).

*BMR* Basal Metabolic Rate, *FM* Fat Mass, *FFM* Fat-Free Mass, Δ Change values from baseline.

^a^Denotes *P* < 0.05 indicating a statistically significant correlation value.

**Fig. 4:**
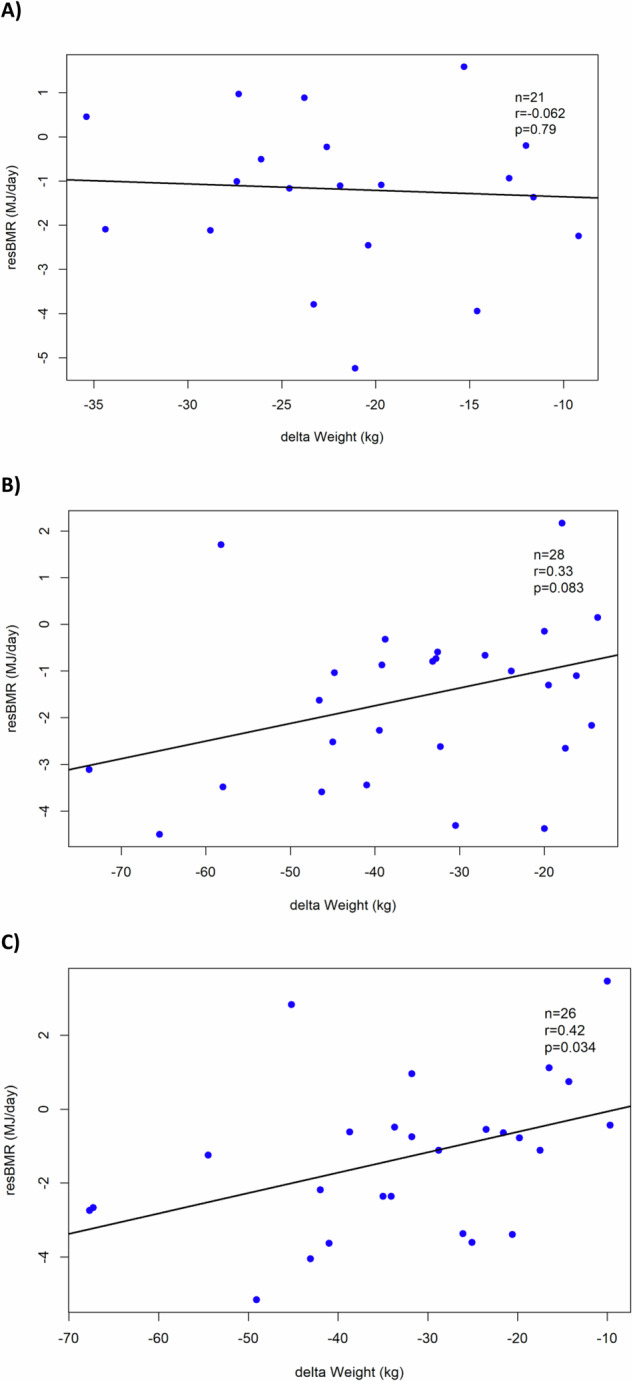
Correlation plots between the extent of metabolic adaptation or residual BMR (resBMR) and changes in body weight in gastric bypass patients. Correlation plot between the extent of resBMR and changes in body weight in gastric bypass patients at **A** 3-, **B** 12- and **C** 24-months post-surgery. resBMR is defined as the difference between the observed BMR (as measured by indirect calorimetry) from the predicted BMR based on the linear regression equations.

Finally, although there was no association between metabolic adaptation and weight changes at 3-months post-surgery (r = −0.062, *p* = 0.79), at 12-months a moderate positive correlation emerged (r = 0.33, *p* = 0.083), and by 24-months it reached statistical significance (r = 0.42, *p* = 0.034) (Fig. 4).

## Discussion

This is the first prospective study to measure BMR and body composition using standardised gold-standard methodology at 3-, 12- and 24-months in patients after GBP surgery and time-matched comparators. It was hypothesized that the expected down regulation of BMR observed after weight-loss, over and above that explained by changes in FM and FFM, would be attenuated in patients 3-months following GBP.

Metabolic adaptation (the change in BMR that is greater than would be predicted from changes in body composition alone during negative energy balance) was observed in patients at 3-months post-surgery when the magnitude of weight loss is greatest (approx. 6.8 kg mean weight loss per month from baseline). This finding is in line with previous studies that observed an adaptive response with approximately 5.5 kg mean weight loss per month at ≤6 months post-surgery [10, 13, 22]. This body composition independent reduction in BMR is hypothesized to be an evolutionary biological process that “slows down metabolism” during periods of food scarcity or significant negative energy balance to increase chances of survival [20, 21]. It appears to be induced by a collection of physiological and neuroendocrine shifts, such as a reduction in plasma insulin levels and associated lower glycogen levels to sustain the brain and body’s energy requirements [23].

At 12-months post-surgery, mean weight loss decreased by 57.4% per month (approx. 2.9 kg mean weight loss per month) and this was also accompanied by a significant degree of metabolic adaptation. This finding is at variance with Knuth et al.’s (2014) data that demonstrated a lack of metabolic adaptation with approx. 3.4 kg mean weight loss per month at 12-months post-surgery. They also used standardised indirect calorimetry to measure absolute BMR and the recommended linear regression method to assess the degree of metabolic adaptation. However, the small sample size (*n* 13) could have driven a statistically nonsignificant result (type 2 error).

Conversely, despite an even smaller sample size (*n* 5), Tam et al. [24] reported the same finding as the present paper where metabolic adaptation was observed at 12-months following GBP. However, they defined metabolic adaptation as a “negative residual value” rather than the recommended approach of assessing whether the residual is significantly different from zero [25]. Therefore, studies with adequate sample sizes to detect clinically relevant differences and appropriate statistical techniques to assess metabolic adaptation are required to confirm that the subsequent decline in BMR was not attributed solely to the reduction in FM and FFM levels 12-months following surgical weight loss.

An interesting pattern emerged in the correlation analysis between the degree of metabolic adaptation and weight loss following GBP. The unexpected absence of a significant association at 3-months post-surgery suggests that metabolic adaptation might not heavily influence immediate weight loss outcomes shortly after the procedure. This could potentially explain why GBP patients continued to experience weight loss success despite the presence of metabolic adaptation that would typically hinder weight loss. It is possible that other factors such as reduced energy intake and physiological and/or psychological adaptations played a more substantial role following GBP [13, 14].

The influence of metabolic adaptation on weight loss progressively became more apparent as a moderate positive correlation emerged at 12 months, which then became statistically significant at 24 months post-surgery. This correlation pattern suggests that metabolic adaptation could have a significant impact on weight changes during the stabilisation phase after GBP and possibly explains the slower weight loss or maintenance observed between 12- and 24-months post-surgery. Understanding this interplay also allows for tailored interventions at 12-months post-surgery to maximise weight loss efforts. Additionally, future research could investigate whether nutrition intervention strategies, such as time-restricted eating or modifying meal composition, can further reduce the impact of this long-term altered metabolic status, potentially improving weight loss and metabolic health.

Nevertheless, it is unclear whether GBP patients maintained weight loss more effectively despite the presence and influence of metabolic adaptation. This is supported by the degree of metabolic adaptation observed in the GBP group being statistically similar to the values obtained by the weight-stable comparator group from 12 months post-surgery suggesting that although metabolic adaptation was present in the surgery group, it appears to be attenuated in the longer-term post-surgery, which may positively impact weight loss and limit weight recidivism.

The underlying biological mechanism of this phenomenon is unclear but it is possible that during a slower rate of weight loss and/or during the weight loss maintenance phase, the therapeutic effects from adipocentric signals such as enhanced leptin sensitivity (owing to significant FM reduction) may aid in attenuating the degree of metabolic adaptation through its actions on triidodothyronine (T3) balance and the mitochondrial content and coupling alterations [26]. The observed increase in overall %FFM per body weight may contribute as well.

A moderate positive correlation was observed between changes in FFM (kg) and changes in absolute BMR at 12- and 24- months post-surgery. Following weight loss, the reduction in mean FFM explains the consequent reduction in absolute BMR values in patients.

Nevertheless, similar to the degree of metabolic adaptation, the mean absolute BMR values were similar between patients and the comparator group post-surgery; despite the comparator group maintaining their weight and FFM levels, suggesting again that the usual compensatory metabolic response which minimises weight loss during periods of energy deficit [27] appears to be blunted in patients following GBP. Similarly, because BMR is also dependent on fat-mass, a positive correlation was observed between changes in FM and BMR at 12- and 24-months post-surgery [25, 28]. As discussed above, the significant reduction in FM levels with concurrent enhanced leptin bioavailability may potentially contribute to attenuating the expected reduction in BMR and metabolic adaptation following surgical weight loss [26].

In summary, these findings underscore the importance of longitudinal assessment in clarifying the correlations and establishing the causal relationship between metabolic adaptation and post-surgery weight loss.

A limitation of the methodology is that the sample size calculation is primarily based on changes in fat intake, potentially limiting its optimisation for the current analysis of metabolic adaptation. To address this concern, retrospective power calculations were conducted based on measured BMR data from the study. These calculations revealed that, despite the final effective sample sizes of 17 and 25 for the comparator and patient groups, respectively (with the largest standard deviations being 2.0 and 2.6 MJ/day, respectively), our study possesses a statistically robust power of 80%. While this level of statistical power is considered moderate, it allows us to detect a mean difference of 2.0 MJ in BMR between the two groups for both the comparator and patient groups at a significance level of 0.05, utilizing a two-sample two-sided Z test. This ensures that even subtle differences in BMR between groups can be confidently identified, thereby enhancing the reliability and validity of our findings. Moreover, this secondary analysis maximises the utility of existing data, offering valuable insights into the metabolic impacts of GBP under residential conditions. It also offers an opportunity for further exploration of the multifaceted effects of the surgery on metabolic adaptation, thereby broadening the scope and relevance of the research.

While this study featured a larger sample size compared to previous studies, it did not randomize or match the comparator group for sex and BMI. However, baseline weight was used as a covariate in our analysis of residual BMR to ensure that any differences in metabolic adaptation are not solely due to the comparator group being significantly leaner (BMI 27.2 (0.94) kg/m^2^) than GBP patients (BMI 45.5 (0.92) kg/m^2^). This approach allowed for a more accurate assessment of the impact of metabolic adaptation independent of differences in body size.

Despite these baseline differences, our findings retain significance, as the GBP group experienced substantial weight loss while the other group remained weight-stable. However, the similar metabolic adaptation observed in both groups from 1-year post-surgery suggests that factors related to the surgery may have attenuated the expected extent of metabolic adaptation typically associated with significant weight loss. Ideally, implementing a weight-matched control group undergoing weight loss through nutrition therapy and maintaining a comparable activity level to the GBP group could have provided deeper insights and isolated the effects of surgical intervention on weight loss outcomes. However, executing such a group can be challenging due to logistical and practical constraints. Therefore, the weight-stable comparator group still provided valuable benefits by enabling a clearer comparison between groups and establishing a stable baseline. This allowed a focused examination of the specific effects of GBP on metabolic adaptation, potentially without the confounding factor of concurrent weight loss in the comparison group.

Finally, despite the absence of physical activity level measurements, BMR measurements within this study were conducted at complete rest >8 h after the last meal to minimize any potential influence of physical activity and meal-induced thermogenesis, which is often considered a study limitation [29]. Additionally, participants engaged solely in sedentary activity while residing in HISU. Therefore, BMR measurement should not be significantly affected by physical activity, except indirectly through changes in body composition.

This study is unique by investigating changes in BMR in GBP patients up to 24-months post-surgery a concurrent weight-stable comparator groupsetting using highly controlled gold-standard protocols. Another strength of this study is the utilization of linear regression analysis to evaluate improvements in metabolic adaptation following weight loss, instead of relying on the ratio method (i.e., BMR/weight). The latter approach could potentially be influenced by changes in the FFM:FM ratio per kilogram of weight following weight loss [7, 10].

Future controlled intervention human studies are required to clarify the kinetic changes in plasma levels of T3, insulin and leptin and its impact on metabolic adaptation during the rapid weight loss and weight maintenance phase following GBP. It might be useful to study metabolic adaptation in associated physiological responses such as heart rate and glomerular filtration rate too [23]. The degree of metabolic adaptation should be assessed using standardised mathematical modelling as discussed above. It is also worthwhile investigating whether standardising the variables used in the linear regression analysis could aid in comparing future studies that investigate metabolic adaptation following GBP. As the highly metabolically active organs and skeletal muscle are considered major sites of metabolic adaptation [30], medical imaging techniques can be used to measure the volume and mass of FFM components i.e. liver that reduces in size significantly following weight loss. A weight-matched control group, losing weight via nutrition therapy and a similar activity level as the GBP group, is recommended albeit difficult to execute. Finally, as inter-individual post-operative weight loss and clinical response vary considerably [4] and remain poorly understood, it should prompt further research in understanding the predictors (neuroendocrine, gender, age, stress, activity level) and mechanisms of metabolic adaptation following weight loss.

In conclusion, the outcomes of this prospective study suggest that metabolic adaptation is present during the rapid weight loss phase (at least 6.9 kg mean weight loss per month) and weight maintenance phase (from 12-months onwards) following GBP. Therefore, the downregulation in BMR was not fully explained by changes in FM and FFM. However, it appears that the degree of metabolic adaptation was attenuated in the surgical group from 12-months onwards and this may potentially contribute to sustained weight loss and limit weight recidivism. Understanding the underlying mechanisms and predictors that attenuate metabolic adaptation following GBP could potentially help the development of treatments to aid weight loss maintenance after non-surgical weight loss or even weight regain after surgery.

## Supplementary information

Supplementary Figures S1-3 & Table S1

## Data Availability

Data described in the manuscript, including de-identified individual participant data, code book, and analytic code will be made available upon request pending application and approval.
