# Supplementary material for: Metabolic adaptation following gastric bypass surgery: results from a 2-year observational study
Source: Int J Obes (Lond). 2024 Sep 3;48(11):1577–86. doi: 10.1038/s41366-024-01585-5 (PMC11502489; doi:10.1038/s41366-024-01585-5)
Supplement: Supplementary file 1 — Supplementary Figures S1-3 & Table S1 [file 41366_2024_1585_MOESM1_ESM.docx]

**Supplementary material: Figures and tables**

**Supplementary figures**

| Figure S1 | Mean absolute changes in anthropometric measures from baseline for patients and comparators at 3- , 12- and 24-months post-surgery. |
| --- | --- |
| Figure S2 | Box plots for weight, body mass index (BMI), fat mass (FM), fat-free mass (FFM) and basal metabolic rate (BMR) at all time-points for patients and comparators |
| Figure S3 | Scatter plot for changes in Basal Metabolic Rate and changes Fat Mass (Patients only) |
| Figure S4 | Scatter plot for changes in Basal Metabolic Rate and changes Fat Mass (Comparators only). |

**Supplementary tables**

| Table S1 | Baseline associations between body composition and basal metabolic rate for patients and comparator group |
| --- | --- |

**A: Changes in body weight**


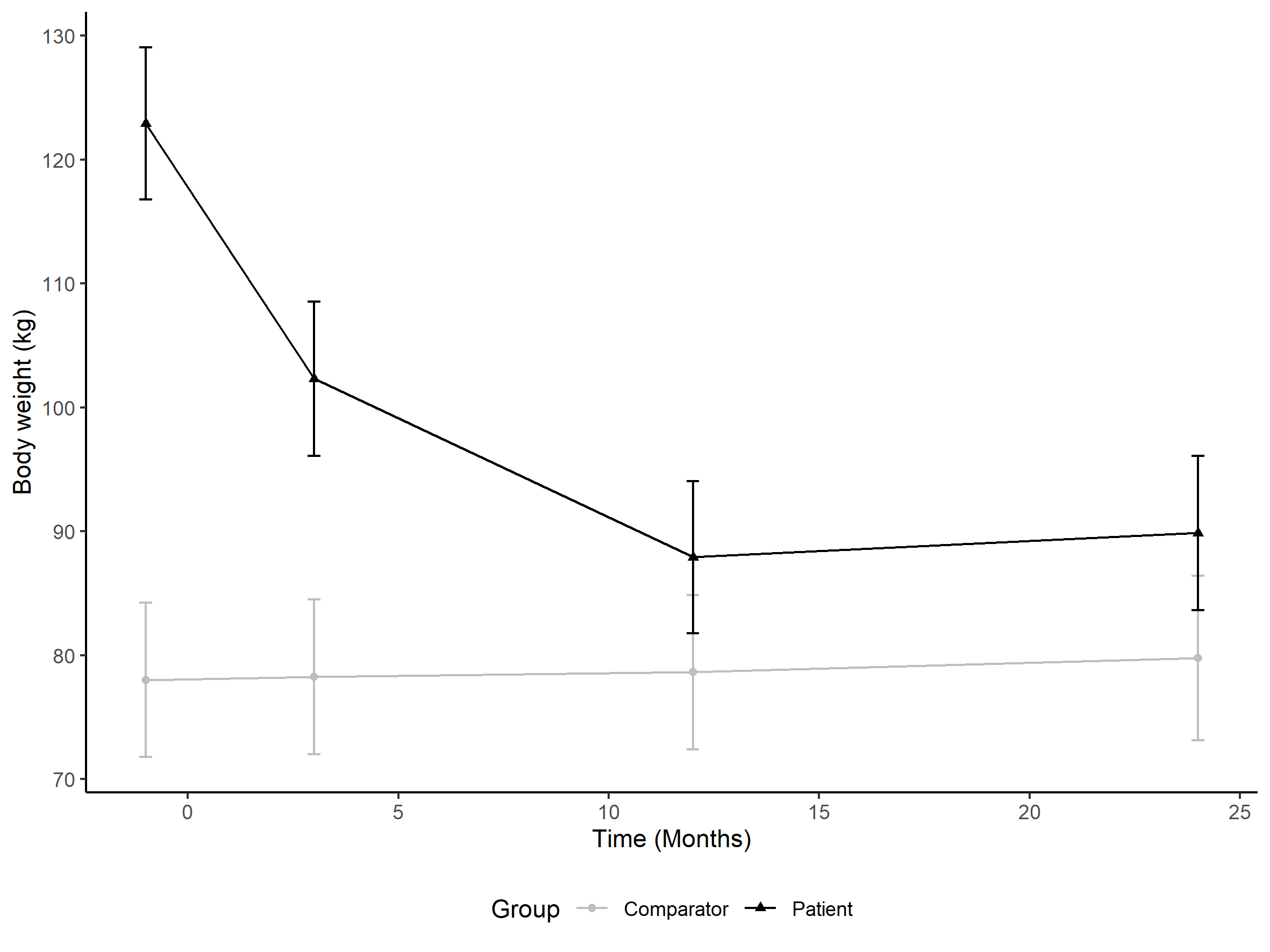


**B: Changes in Body Mass Index (BMI)**


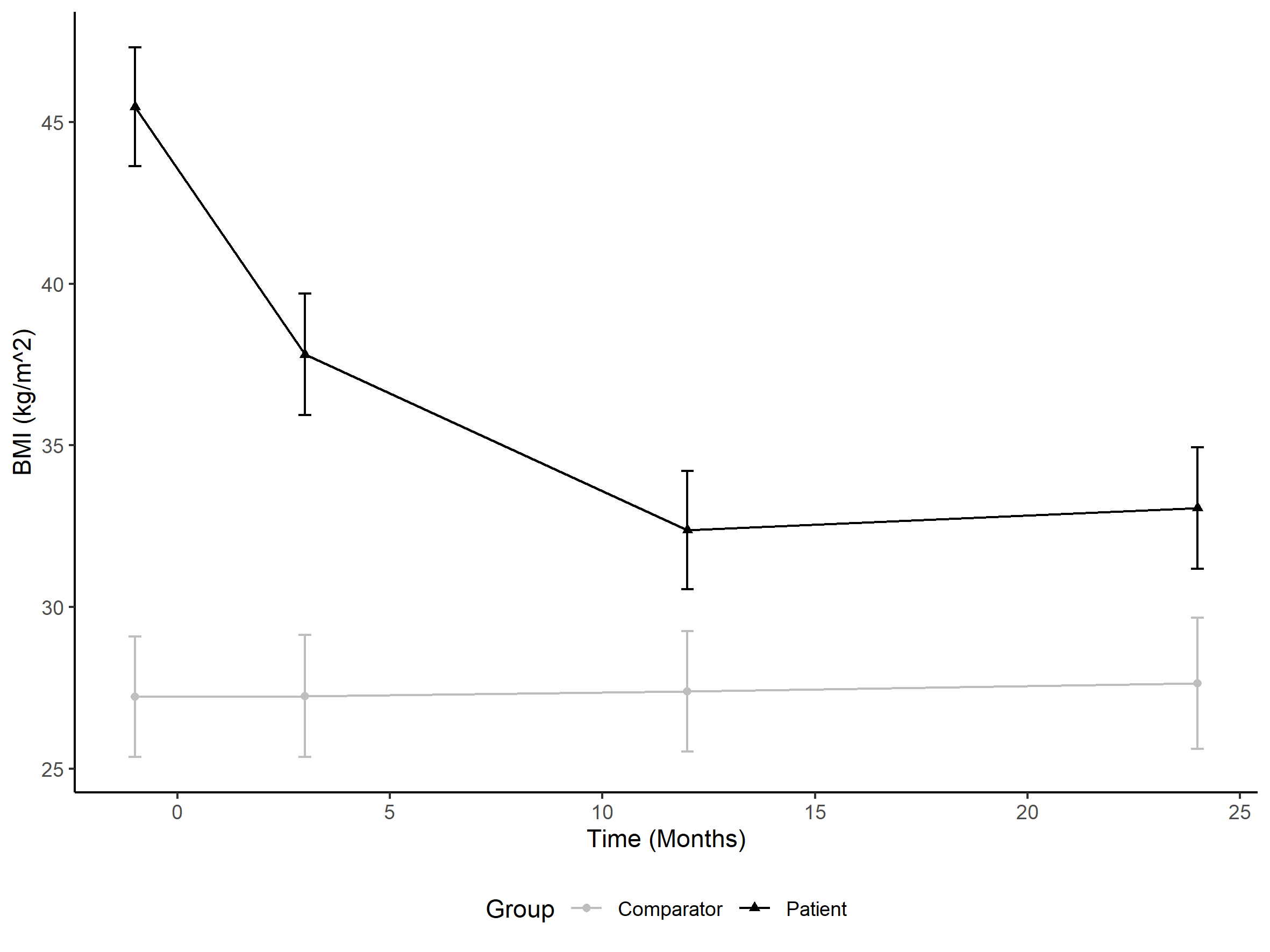


**C: Changes in fat mass (FM)**


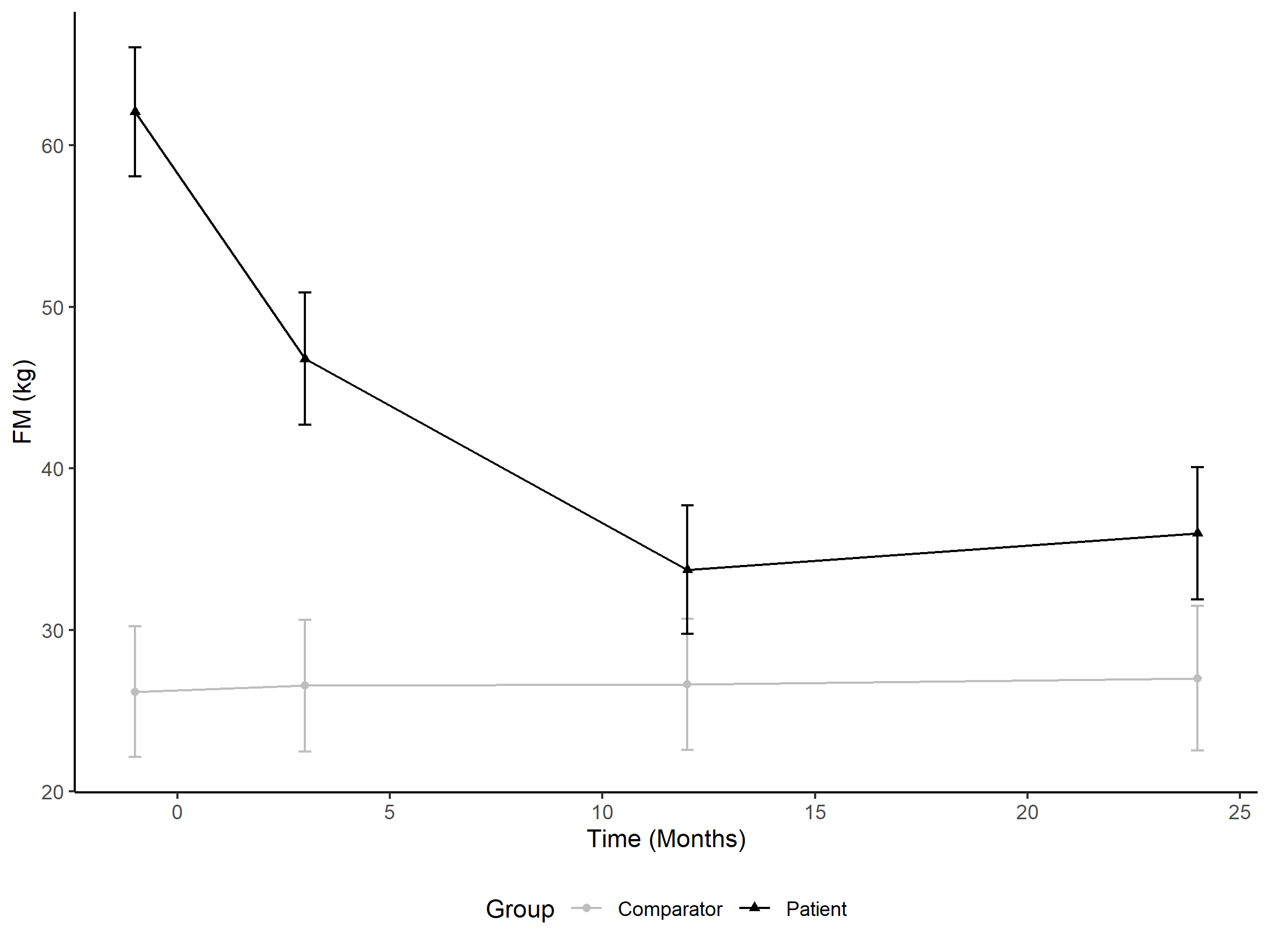


**D: Changes in fat-free mass (FFM)**


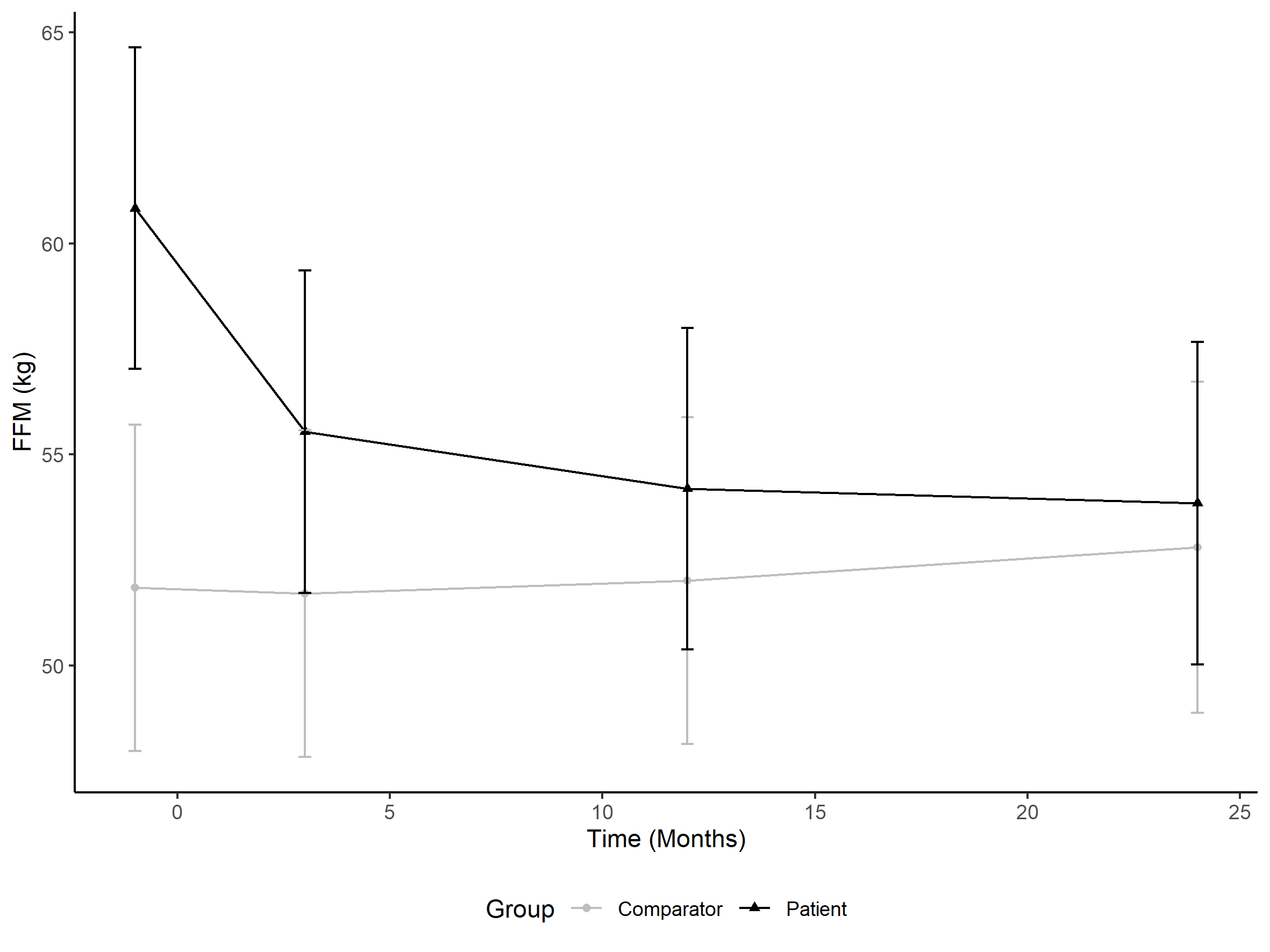


**E: Changes in visceral adipose tissue (VAT)**


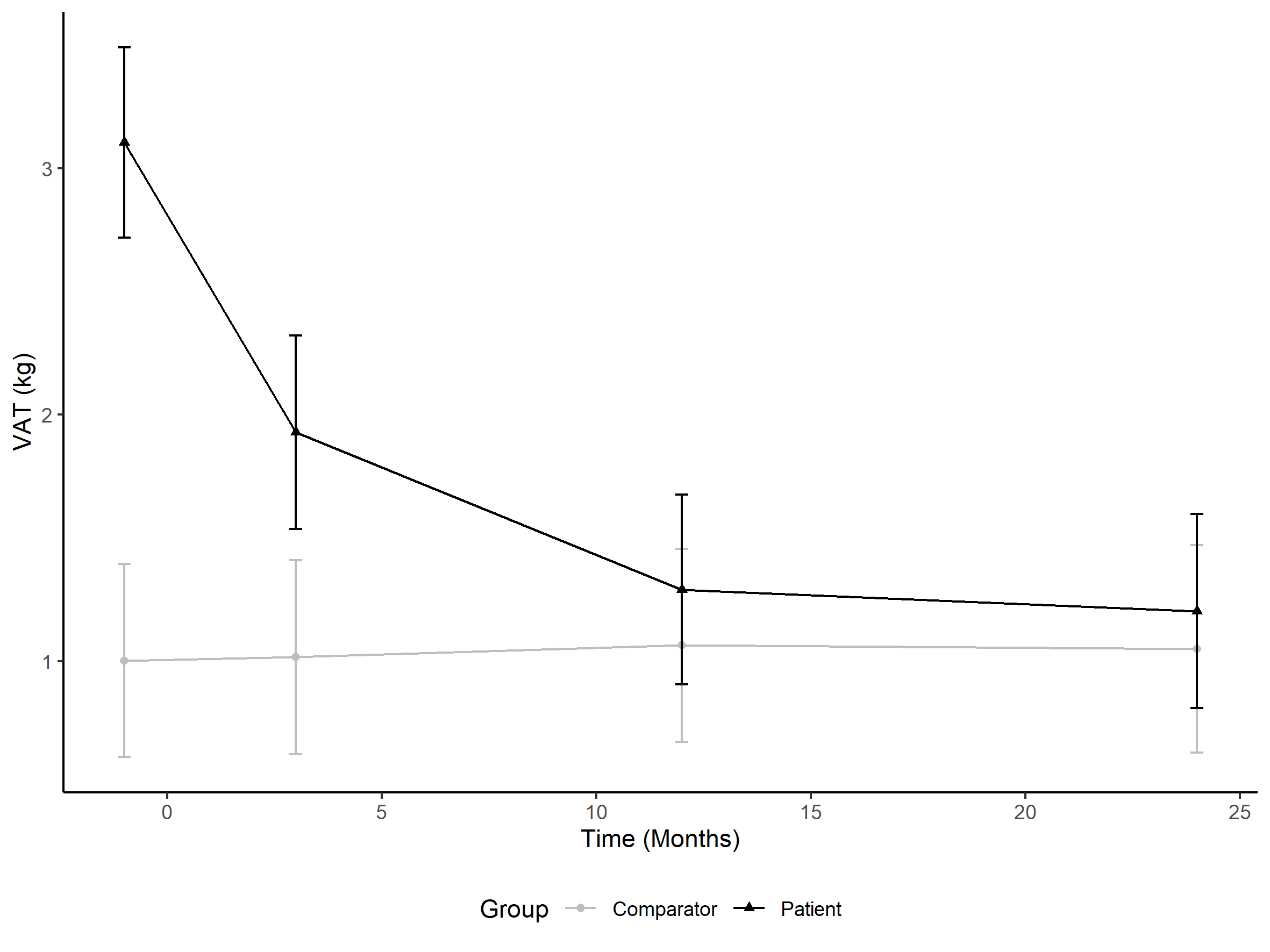


**F: Changes in subcutaneous adipose tissue (SAT)**


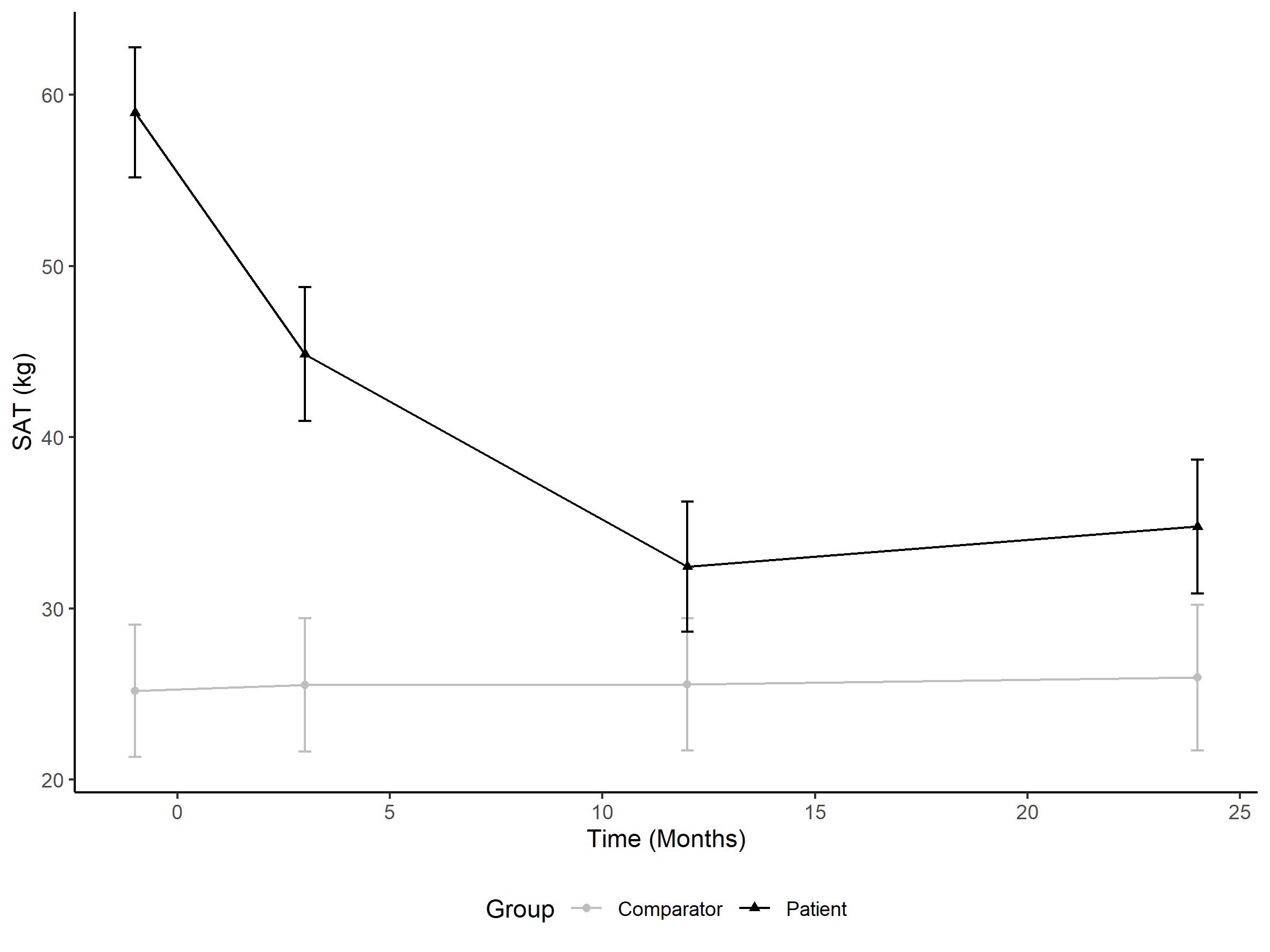


**Figure S1:** Temporal profile in anthropometric measures based on linear mixed model estimates. The error bars represent 95% Confidence intervals. A)Total body weight, B)Body Mass Index, C)Fat Mass, D) Fat-free mass, E)Visceral adipose tissue, F) Subcutaneous adipose tissue for patients and comparator group from baseline (1-month pre-surgery) to 3-, 12- and 24-months post-surgery.


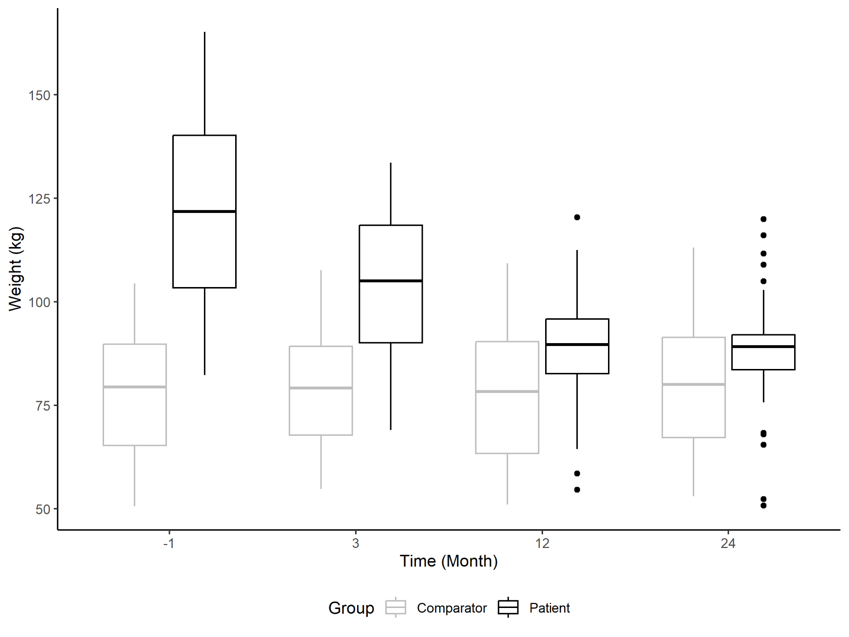

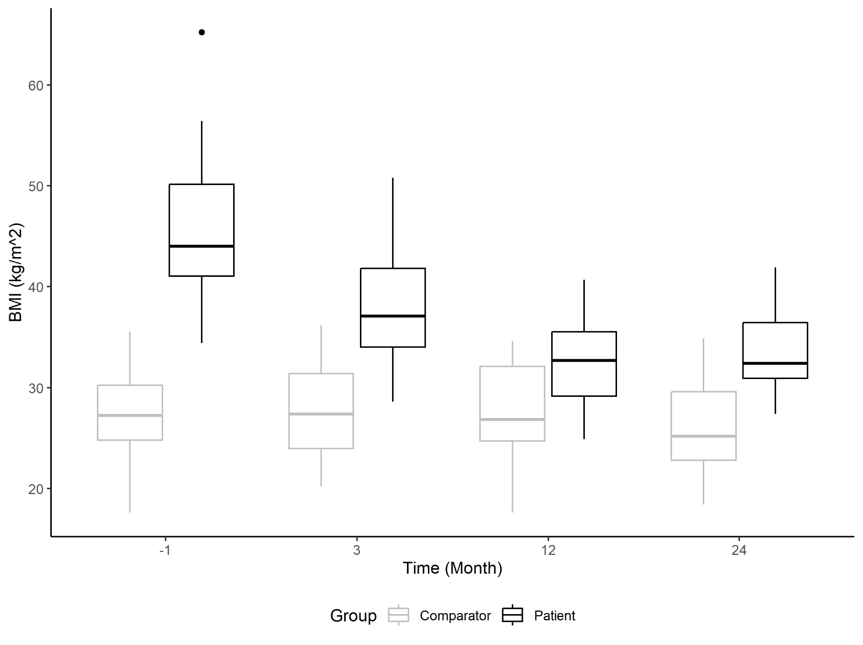


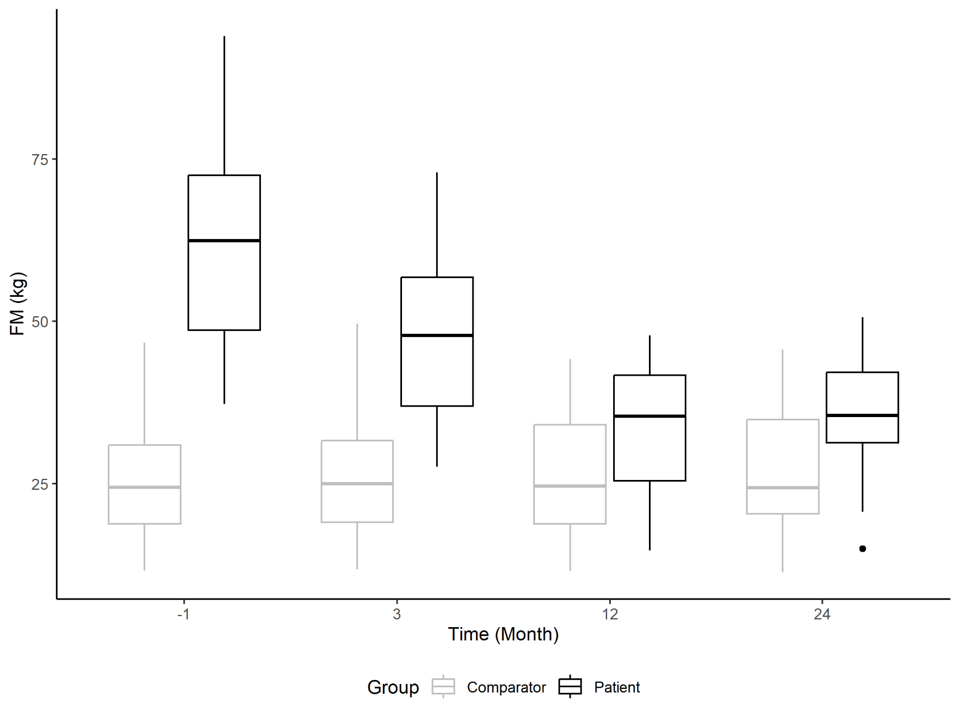

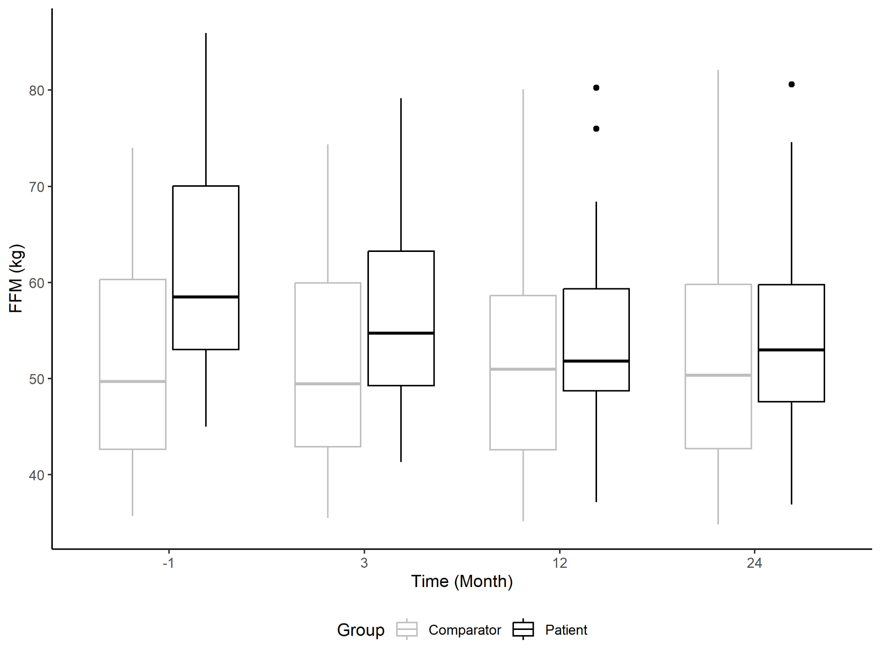


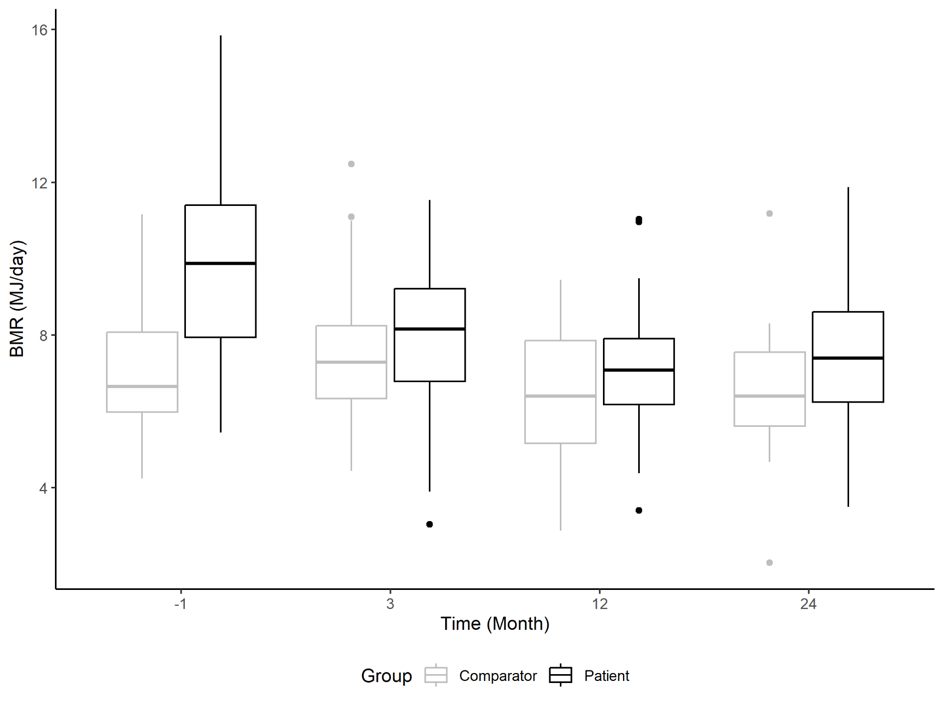


**Figure S2:** Box plots for weight, body mass index (BMI), fat mass (FM), fat-free mass (FFM) and basal metabolic rate (BMR) at all time-points for patients and comparators. The box plots were created in the style of Tukey using the R package ggplot2 with default settings. A boxplot displays the distribution of a continuous variable. It visualises five summary statistics (the median, two hinges and two whiskers), and all "outlying" points individually. The upper whisker extends from the hinge to the largest value no further than 1.5 * IQR from the hinge (where IQR is the inter-quartile range, or distance between the first and third quartiles). The lower whisker extends from the hinge to the smallest value at most 1.5 * IQR of the hinge. Data beyond the end of the whiskers are called "outlying" points and are plotted individually. N values for weight, BMI, FM and FFM: comparator group time point (TP)1: 30, TP2:29, TP3:30, TP4:30; patients TP1: 31,TP2:26, TP3:31, TP4:31. N values for BMR: comparator group TP1:25, TP2:30, TP3:26, TP4:20; patient TP1:27, TP2:22, TP3: 29, TP4:27


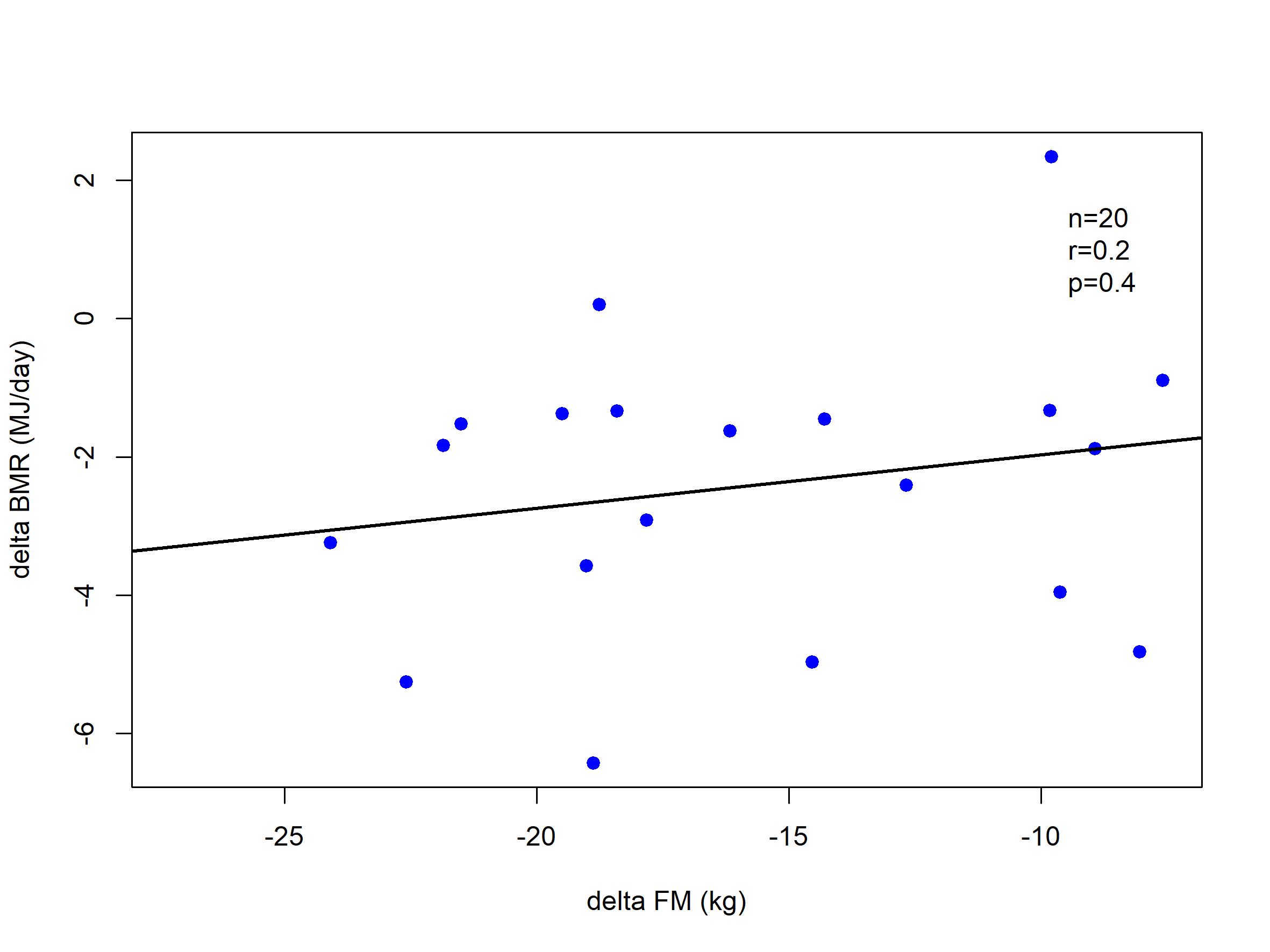


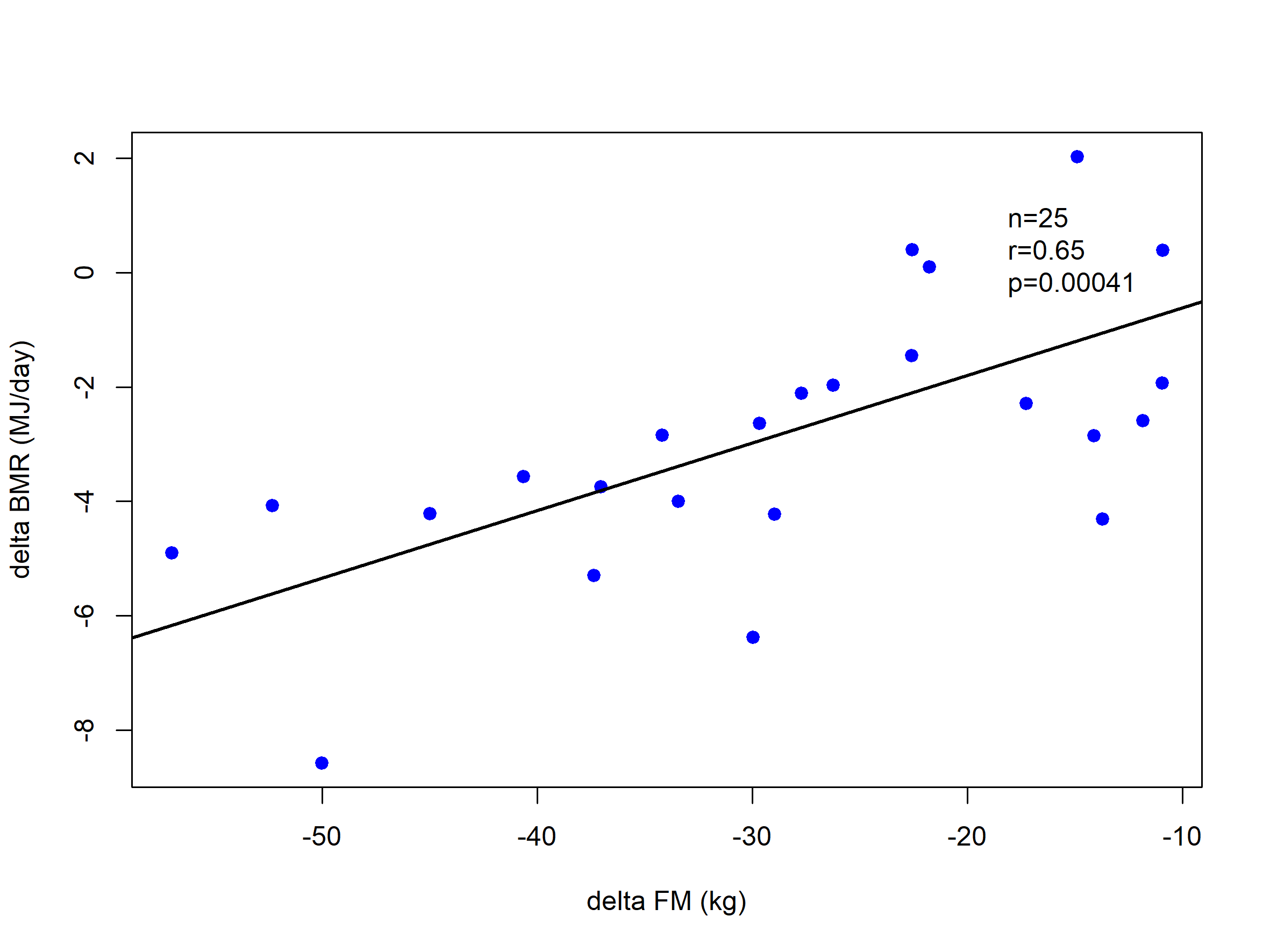


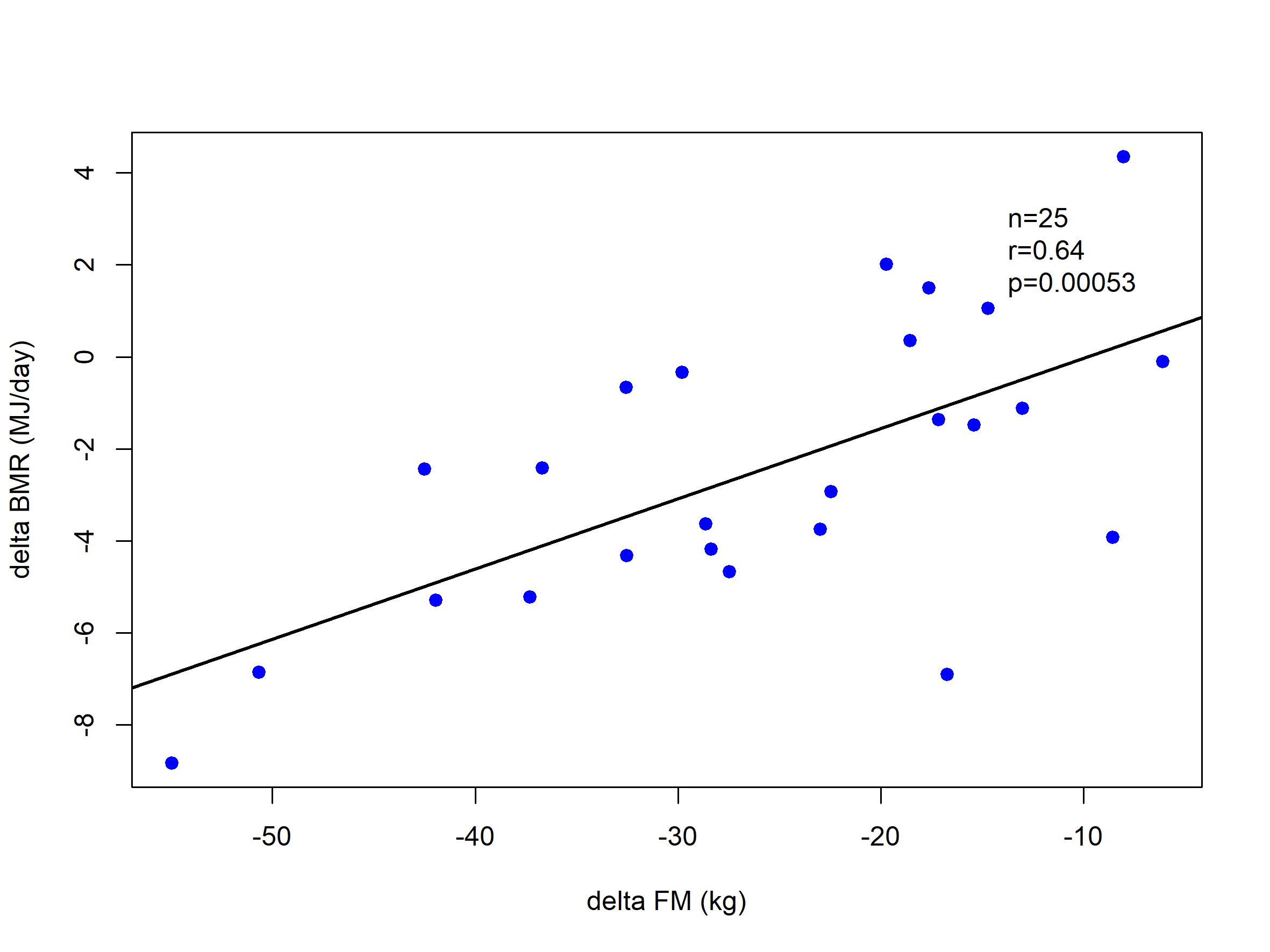


**Figure S3:**  Scatter plot for changes in Basal Metabolic Rate and changes in Fat Mass from baseline at A) 3-months, B) 12- and C) 24-months post-surgery (Patients only). The line is the simple linear regression line; r is the Pearson’s correlation coefficient; p value for the Pearson correlation test.


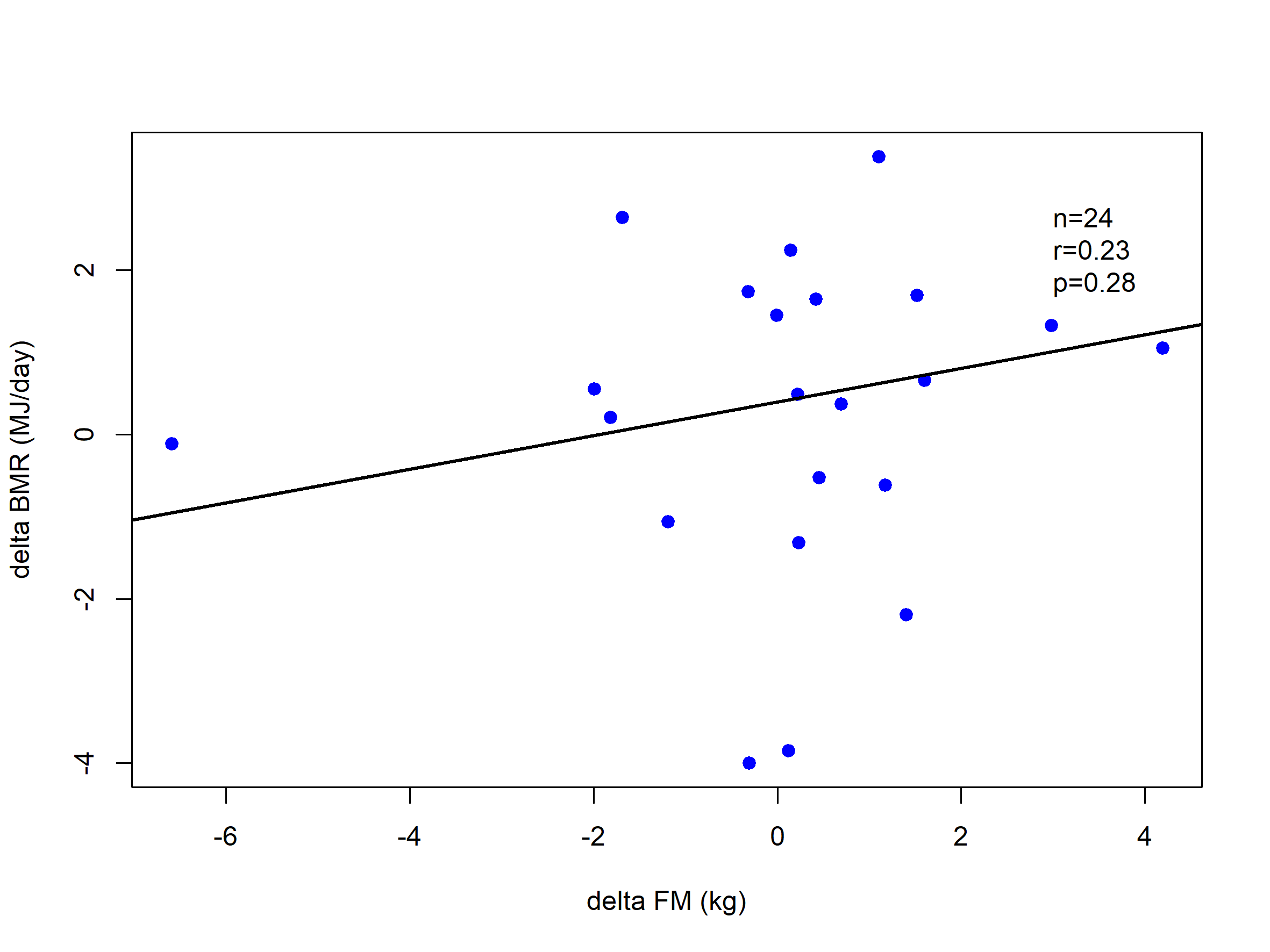


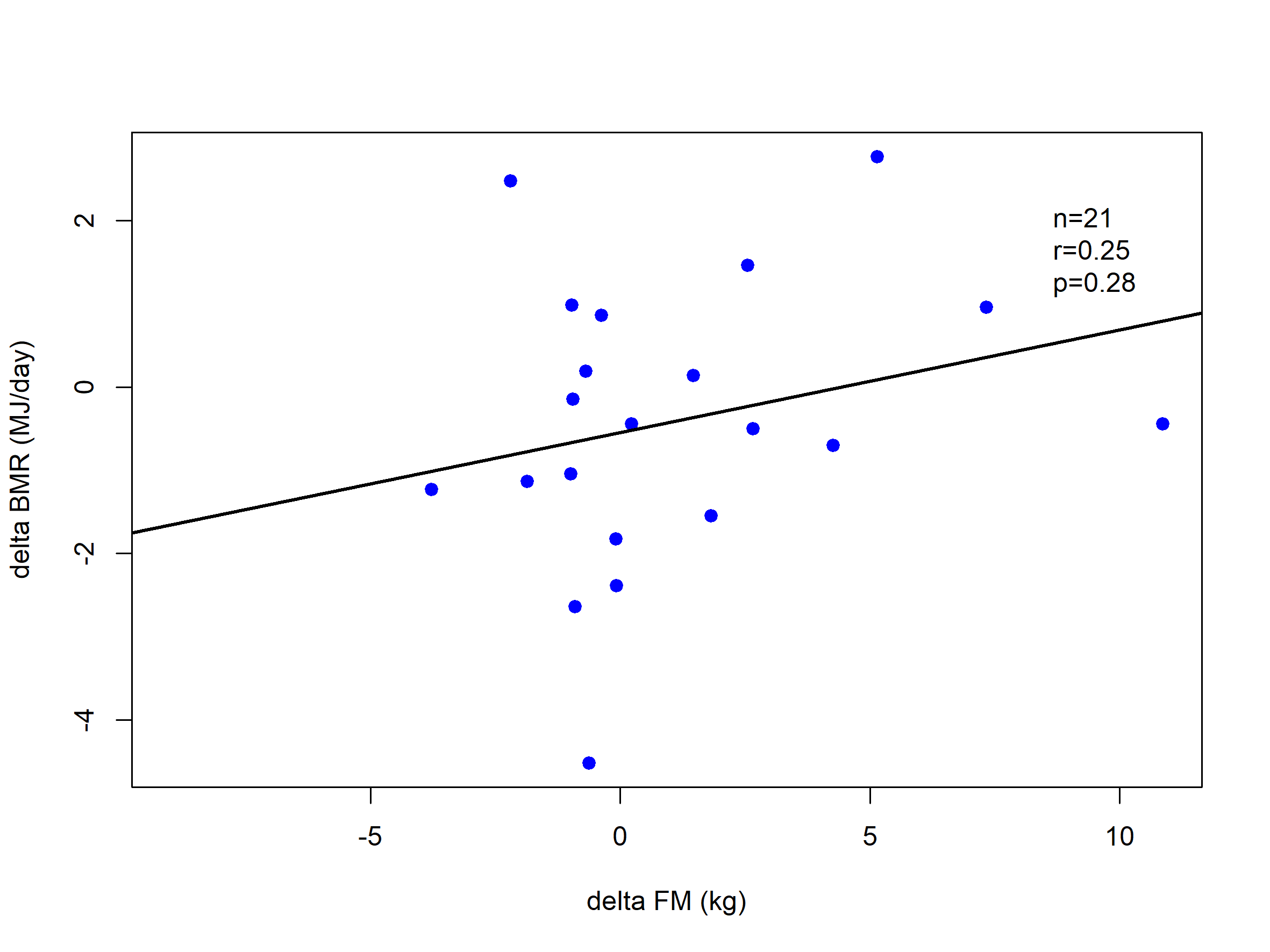


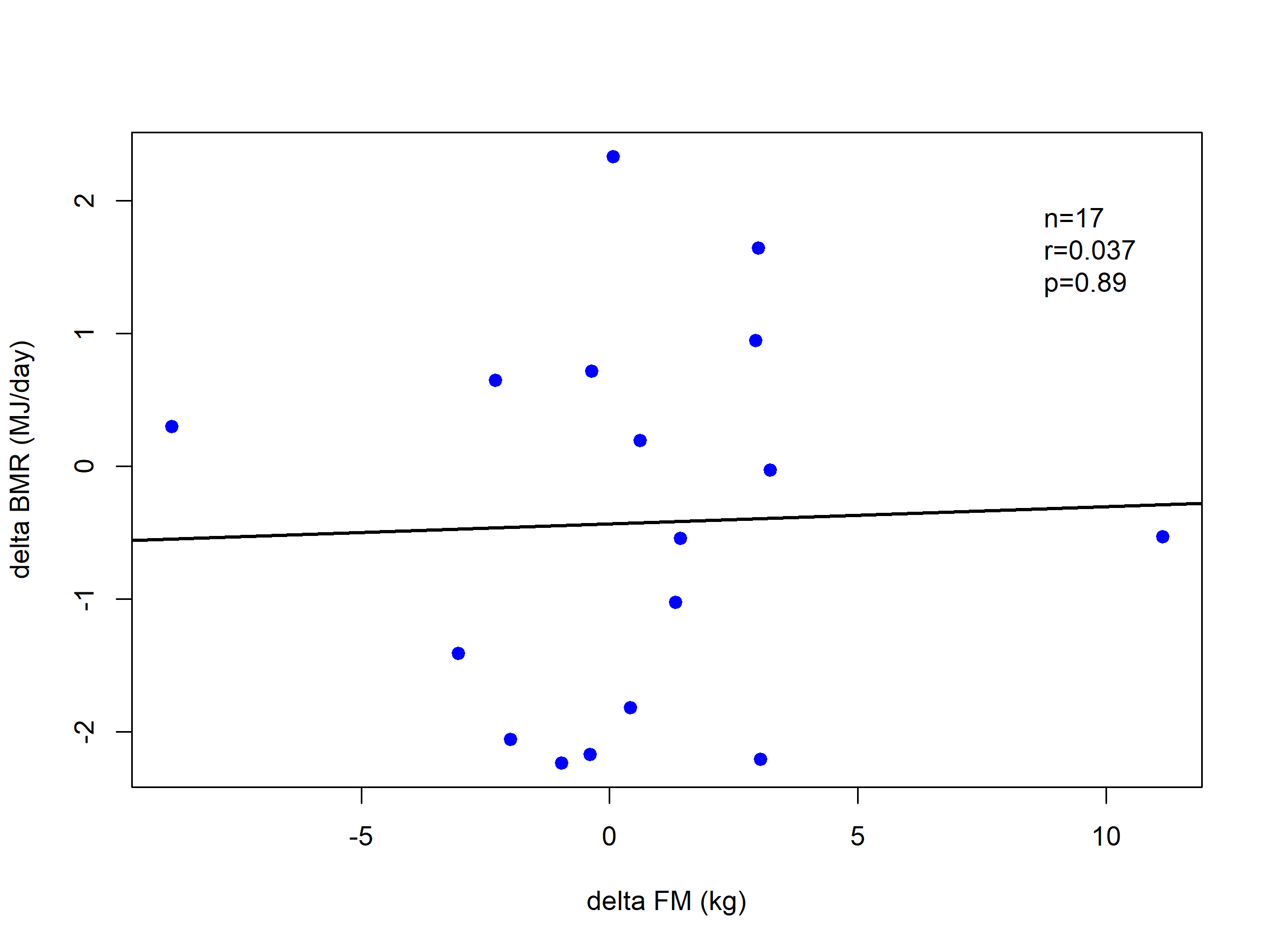


**Figure S4:** Scatter plot for changes in Basal Metabolic Rate and changes in Fat Mass from baseline at A) 3-months, B) 12- and C) 24-months post-surgery (Comparators only). The line is the simple linear regression line; r is the Pearson’s correlation coefficient; p value for the Pearson correlation test.

**Table S1.** Baseline associations between body composition and basal metabolic rate for patients and comparator group

.

|  | **Patients (n 27)** | **Comparator group (n 25)** |
| --- | --- | --- |
|  | BMR (MJ/day) | BMR (MJ/day) |
| Weight (kg) | 0.51 ^a^  (95%CI: 0.17 to 0.75; p=0.01) | 0.39  (95%CI: -0.01 to 0.68; p=0.05) |
| Fat mass (kg) | 0.45 ^a^  (95%CI:0.08 to 0.71;p=0.02) | -0.08  (95%CI:-0.46 to 0.32;p=0.69) |
| Fat-free mass (kg) | 0.47 ^a^  (95%CI:0.11 to 0.72;p=0.01) | 0.61 ^a^  (95%CI:0.29 to 0.81; p=0.0012) |

Associations analysed using Pearson’s correlation. Data presented as r (correlation coefficient) with 95% confidence interval (CI). **a** denotes P<0.05 indicating a statistically significant correlation value. *BMR* Basal Metabolic Rate.
